# Intravenous ferric carboxymaltose in heart failure with iron deficiency (FAIR‐HF2 DZHK05 trial): Sex‐specific outcomes

**DOI:** 10.1002/ejhf.3742

**Published:** 2025-07-31

**Authors:** Mahir Karakas, Tim Friede, Javed Butler, Khawaja M. Talha, Marius Placzek, Thomas Asendorf, Monika Diek, Anna Nosko, Adriane Stas, Stefan Kluge, Dominik Jarczak, Geraldine DeHeer, Meike Rybczynski, Antoni Bayes‐Genis, Michael Böhm, Andrew J.S. Coats, Frank Edelmann, Gerasimos Filippatos, Gerd Hasenfuß, Wilhelm Haverkamp, Mitja Lainscak, Ulf Landmesser, Iain C. Macdougall, Bela Merkely, Burkert M. Pieske, Fausto J Pinto, Tienush Rassaf, Jennifer K. Visser‐Rogers, Giuseppe Rosano, Maurizio Volterrani, Stephan von Haehling, Markus S. Anker, Wolfram Doehner, Hüseyin Ince, Friedrich Koehler, Gianluigi Savarese, Muhammad Shahzeb Khan, Ursula Rauch Kröhnert, Tommaso Gori, Teresa Trenkwalder, Ibrahim Akin, Christina Paitazoglou, Iwona Kobielusz‐Gembala, Luca Kuthi, Norbert Frey, Manuela Licka, Stefan Kääb, Karl‐Ludwig Laugwitz, Piotr Ponikowski, Stefan D. Anker

**Affiliations:** ^1^ Department of Intensive Care Medicine University Medical Centre Hamburg‐Eppendorf Hamburg Germany; ^2^ Partner Site Hamburg/Kiel/Lübeck German Centre for Cardiovascular Research (DZHK) Hamburg Germany; ^3^ Department of Medical Statistics University Medical Centre Göttingen Göttingen Germany; ^4^ Partner site Lower Saxony German Centre for Cardiovascular Research (DZHK) Göttingen Germany; ^5^ Department of Medicine University of Mississippi Medical Centre Jackson MS USA; ^6^ Baylor Scott and White Research Institute Dallas TX USA; ^7^ Department of Cardiovascular Disease Loyola University Medical Centre Maywood IL USA; ^8^ Deutsches Herzzentrum der Charité, Campus Virchow Klinikum Berlin, Germany; Institute of Health Centre for Regenerative Therapies (BCRT), German Centre for Cardiovascular Research (DZHK) partner site Berlin, Charité Universitätsmedizin Berlin Germany; ^9^ Department of Medical Informatics University Medical Centre Göttingen Göttingen Germany; ^10^ Department of Cardiology University Heart & Vascular Centre Hamburg, University Medical Centre Hamburg‐Eppendorf Hamburg Germany; ^11^ Partner Site Hamburg/Kiel/Lübeck, German Centre for Cardiovascular Research (DZHK) Hamburg Germany; ^12^ Heart Institute Hospital Universitari Germans Trias i Pujol Barcelona Spain; ^13^ Department of Medicine III and HOMICAREM (Homburg Institute for CardioRenalMetabolic Medicine) Saarland University Homburg Germany; ^14^ Heart Research Institute Sydney NSW Australia; ^15^ Department of Cardiology, Angiology and Intensive Care Medicine Deutsches Herzzentrum der Charité, Campus Virchow Klinikum Berlin Germany; ^16^ German Centre for Cardiovascular Research (DZHK) Partner site Berlin, Charité Universitätsmedizin Berlin Germany; ^17^ Department of Cardiology Attikon University Hospital, School of Medicine, National and Kapodistrian University of Athens Athens Greece; ^18^ Department of Cardiology and Pneumology University Medical Centre Göttingen, Georg August University of Göttingen Göttingen Germany; ^19^ Division of Cardiology General Hospital Murska Sobota Murska Sobota Slovenia; ^20^ Faculty of Medicine University of Ljubljana Ljubljana Slovenia; ^21^ Department of Cardiology, Angiology and Intensive Care Medicine Deutsches Herzzentrum der Charité, Campus Benjamin Franklin Berlin Germany; ^22^ Berlin Institute of Health Center for Regenerative Therapies (BCRT) Berlin Germany; ^23^ DZHK (German Centre for Cardiovascular Research), Partner site Berlin Berlin Germany; ^24^ Department of Renal Medicine King's College Hospital London UK; ^25^ Heart and Vascular Centre Semmelweis University Budapest Hungary; ^26^ Division of Cardiology, Department of Internal Medicine University Medicine Rostock Rostock Germany; ^27^ Centro Academico de Medicina de Lisboa, CCUL@RISE Faculdade de Medicina da Universidade de Lisboa Lisbon Portugal; ^28^ West German Heart‐ and Vascular Centre, Department of Cardiology and Vascular Medicine University Hospital Essen, University Duisburg‐Essen Essen Germany; ^29^ Coronado Research Newcastle UK; ^30^ San Raffaele Open University of Rome Rome Italy; ^31^ Cardiology San Raffaele Cassino Hospital Cassino Italy; ^32^ IRCCS San Raffaele Roma Roma Italy; ^33^ Cardiology Clinical Academic Group, Molecular and Clinical Sciences Research Institute City St George's, University of London London UK; ^34^ IRCCS San Raffaele Rome Rome Italy; ^35^ San Raffaele Open University in Rome Rome Italy; ^36^ Berlin Institute of Health – Centre for Regenerative Therapies, and Department of Cardiology (CVK), Deutsches Herzzentrum der Charité and German Centre for Cardiovascular Research Partner Site Berlin Charité – Universitätsmedizin Berlin Berlin Germany; ^37^ Deutsches Herzzentrum der Charité (DHZC), Department of Cardiology, Angiology and Intensive Care Medicine Campus Charité Mitte Berlin Germany; ^38^ Centre for Cardiovascular Telemedicine Charité – Universitätsmedizin Berlin, Corporate Member of Freie Universität Berlin and Humboldt‐Universität zu Berlin Berlin Germany; ^39^ DZHK (German Centre for Cardiovascular Research), Partner Site Berlin Berlin Germany; ^40^ Department of Clinical Science and Education Karolinska Institutet Stockholm Sweden; ^41^ The Heart Hospital Baylor Scott and White Health Plano TX USA; ^42^ Department of Cardiology, Cardiology I University Medical Centre Mainz Mainz Germany; ^43^ German Centre for Cardiovascular Research (DZHK), Standort RheinMain Frankfurt Germany; ^44^ School of Medicine and Health, Department of Cardiovascular Diseases Technical University of Munich, German Heart Centre Munich, TUM University Hospital Munich Germany; ^45^ Partner Site Munich Heart Alliance German Centre for Cardiovascular Research Munich Germany; ^46^ Department of Cardiology, Angiology, Haemostaseology and Medical Intensive Care University Medical Centre Mannheim, Medical Faculty Mannheim, Heidelberg University Heidelberg Germany; ^47^ Partner Site, Heidelberg/Mannheim DZHK (German Centre for Cardiovascular Research (DZHK)) Mannheim Germany; ^48^ Department of Cardiology, Angiology and Intensive Care Medicine University Heart Centre Lübeck, Medical Clinic II, University Hospital Schleswig‐Holstein Lübeck Germany; ^49^ Oświęcimskie Centrum Badań Klinicznych Oświęcim Poland; ^50^ University Hospital Heidelberg, Department of Cardiology, Angiology and Pneumolgy, Clinical Trial Unit Heidelberg, Germany; DZHK (German Centre for Cardiovascular Research (DZHK)), Partner Site, Heidelberg/Mannheim Mannheim Germany; ^51^ LMU University Hospital, LMU Munich Department of Medicine I Munich Germany; ^52^ Department of Internal Medicine I Technical University Munich University Hospital Munich Germany; ^53^ Partner Site Munich Heart Alliance DZHK (German Centre for Cardiovascular Research) Munich Germany; ^54^ Institute of Heart Diseases Medical University and University Hospital Wroclaw Poland

**Keywords:** Sex‐specific outcomes, Heart failure, Iron deficiency, Ferric carboxymaltose, Randomized controlled clinical trials

## Abstract

**Aims:**

Intravenous iron has emerged as a guideline‐recommended therapy in patients with heart failure and iron deficiency, but the potential sex‐related differences in efficacy are unknown. We aimed to assess sex‐specific outcomes in the Intravenous Iron in Patients with Systolic Heart Failure and Iron Deficiency to Improve Morbidity & Mortality (FAIR‐HF2‐DZHK05) trial.

**Methods and results:**

FAIR‐HF2 included 1105 heart failure patients with a left ventricular ejection fraction ≤45% and iron deficiency. A total of 368 women (mean age 68.7 ± 13.0 years) and 737 men (mean age 70.5 ± 11.0 years) were randomized to intravenous ferric carboxymaltose or placebo. The three primary endpoints were (i) time to cardiovascular death or first heart failure hospitalization, (ii) total heart failure hospitalizations, and (iii) time‐to‐first event of cardiovascular death or heart failure hospitalization only in patients with transferrin saturation <20% at baseline. The hazard ratio (HR) for the first primary outcome was 1.07 (95% confidence interval [CI] 0.63–1.82, *p* = 0.80) in women and 0.74 (95% CI 0.57–0.95, *p* = 0.016) in men, while the rate ratios (RRs) for the second primary outcome were 1.06 (95% CI 0.55–2.05, *p* = 0.86) and 0.79 (95% CI 0.58–1.08, *p* = 0.136), respectively, and the HRs for the third primary outcome event were 1.21 (95% CI 0.62–2.36, *p* = 0.58) and 0.73 (95% CI 0.55–0.97, *p* = 0.028), respectively. Regarding safety outcomes, the HR for all‐cause mortality was 1.46 (95% CI 0.78–2.76, *p* = 0.24) in women, suggesting increased mortality risk under iron supplementation, in contrast to 0.86 (95% CI 0.64–1.16, *p* = 0.33) in men (*p* for interaction = 0.13).

**Conclusions:**

This analysis indicates relevant differential efficacy of intravenous iron in heart failure across both sexes. While men receiving ferric carboxymaltose experienced a clinically relevant reduction in cardiovascular death and heart failure hospitalizations, women did not derive similar benefits. The results are clinically relevant and prompt validation in other large outcome trials of intravenous iron supplementation in heart failure.

Clinical Trial Registration: ClinicalTrials.gov NCT03036462.

## Introduction

Iron deficiency correlates with disease severity of heart failure and is associated with impaired symptomatic and functional outcomes, as well as with increased mortality.^1^ Since several large outcome trials have shown that intravenous iron supplementation in these patients is beneficial with regard to prognostic outcomes, intravenous iron supplementation has emerged as a guideline‐recommended therapy in patients with heart failure and concomitant iron deficiency.^2^, ^3^

Clinical and experimental evidence suggests that oestrogen manipulates intracellular iron metabolism and that elevated levels of oestrogen associate with increased systemic iron availability, thereby potentially altering the therapeutic response to intravenous iron in women.^4^ However, data on sex‐specific outcomes are scarce. Out of the three randomized outcome trials, the IRONMAN (Intravenous Ferric Derisomaltose in Patients with Heart Failure and Iron Deficiency) trial did not report relevant differential efficacy in men and women.^5^ The AFFIRM‐AHF (A Randomised, Double‐blind Placebo Controlled Trial Comparing the Effect of Intravenous Ferric Carboxymaltose on Hospitalizations and Mortality in Iron Deficient Subjects Admitted for Acute Heart Failure) trial reported a significant reduction in heart failure hospitalizations in a COVID adjusted analysis in patients with worsening heart failure treated with ferric carboxymaltose, but with a relative risk reduction for the primary endpoint of 36% in men (rate ratio [RR] 0.64, 95% confidence interval [CI] 0.46–0.89) in contrast to a numerically increased relative risk of 5% (RR 1.05, 95% CI 0.72–1.53) in women.^6^ Similarly, the HEART‐FID (Ferric Carboxymaltose in Heart Failure with Iron Deficiency) trial, which failed to show a significant benefit of intravenous iron supplementation in patients with chronic stable heart failure, indicated differential efficacy for men and women in its basic serial subgroup analysis, with a hazard ratio (HR) for the main secondary endpoint, a composite of cardiovascular death or first hospitalization for heart failure, of 0.86 (95% CI 0.74–1.00) in men and 1.11 (95% CI 0.86–1.43) in women.^7^

The FAIR‐HF2 (Intravenous Iron in Patients with Systolic Heart Failure and Iron Deficiency to Improve Morbidity & Mortality) trial was an investigator‐initiated multicentre randomized trial designed to assess the benefits and risks of intravenous iron therapy in patients with heart failure and concomitant iron deficiency.^8^, ^9^ We conducted a pre‐specified in‐depth sex‐specific analysis in FAIR‐HF2 comparing the efficacy and safety of ferric carboxymaltose therapy in women versus men.

## Methods

### Study design

FAIR‐HF2 was a prospective, randomized, double‐blind, investigator‐initiated, multicentre trial in patients with chronic stable heart failure and iron deficiency. The design of the FAIR‐HF2 trial has been recently published.^10^ In summary, between March 2017 and November 2023, 1824 patients were screened, and 1105 participants were randomized at 70 sites in six countries. Patients with chronic heart failure and reduced ejection fraction of at least 3‐month duration with left ventricular ejection fraction ≤45% and evidence of serum iron deficiency (serum ferritin <100 ng/ml or between 100 ng/ml and 299 ng/ml, provided in these patients the transferrin saturation is <20%) were randomized 1:1 to intravenous ferric carboxymaltose or placebo, and were followed for a median of 16.6 months. Study treatment dosing phases were divided into a repletion and a maintenance phase. Up to a maximum of 2000 mg ferric carboxymaltose was given during the first two visits at baseline and at week 4, which was then followed by fixed maintenance doses of 500 mg that were administered every 4 months unless haemoglobin exceeded 16 g/dl or serum ferritin exceeded 800 ng/ml. Patients assigned to the placebo group received a saline administration at all visits.

### Outcomes and assessment

The three primary endpoints were as follows: (i) time‐to‐first event of either cardiovascular death or heart failure hospitalization; (ii) composite rate of first and recurrent heart failure hospitalizations; and (iii) time‐to‐first event of either cardiovascular death or heart failure hospitalization in the subgroup of patients with a transferrin saturation <20% at baseline. All reported primary endpoints were adjudicated by an independent endpoint committee blinded to randomized treatment assignment. Adverse events (AEs) were also monitored and reported in a blinded manner. Secondary endpoints included the change in New York Heart Association (NYHA) functional class, the change in EuroQol‐5 Dimension (EQ‐5D) score, and the change in 6‐min walk distance all from baseline to 12 months, as well as the change in patient‐reported global assessment of subjective well‐being score during follow‐up until 12 months. Safety endpoints included all‐cause mortality and cardiovascular mortality at 3 years of follow‐up.

### Statistical analysis

All analyses were performed separately in women and men as pre‐specified in the trial design. Wilcoxon two‐sample tests for continuous variables and Chi‐squared tests for categorical variables were used to compare the distribution of risk factors and levels of biomarkers across the two randomized treatment arms in sex‐specific analyses. The analysis of the primary endpoints related to time‐to‐first cardiovascular death or heart failure hospitalization (in the full population and in the population of patients with transferrin saturation <20% at baseline) was performed using the Cox proportional hazard model to derive HR with 95% CI. The analysis for the primary endpoint of total (first and recurrent) heart failure hospitalization was based on the semi‐parametric regression model for the mean and rate functions of recurrent events proposed by Lin and colleagues (LWYY) and reported as RR and 95% CI. To assess the interaction of treatment and sex, the corresponding interaction term was included in the regression models (Cox regression, LWYY model). Recurrent event and time to event outcomes were illustrated by cumulative incidence functions and Kaplan–Meier curves stratified by treatment group, respectively. The Hochberg procedure was utilized to analyse the three primary hypotheses to control the familywise type 1 error rate at the pre‐specified level of two‐sided 0.05.^11^ The familywise type I error rate across the four secondary endpoints, which is formally tested only if all primary hypotheses are rejected, was also controlled using the Hochberg procedure. Statistical analyses were performed using the R software, version 4.3.1 (R Foundation for Statistical Computing, Vienna, Austria).

## Results

**Figure 1:**
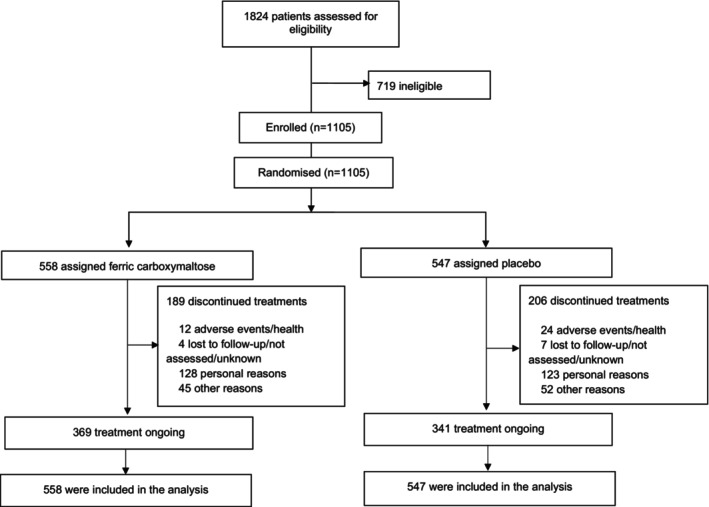
Consort diagram.

The baseline characteristics are shown in *Table* *1* and online supplementary *Table* Appendix *S1*. A total of 1105 participants were randomized, and 558 patients were assigned to the ferric carboxymaltose group and 547 to the placebo group (*Figure* *1*). Women comprised one third of the study population (*n* = 368). The mean age of patients that were women was 69 ± 13 years, while the mean age of men was 71 ± 11 years. Men had clearly higher rates of diabetes (49% vs. 38%, *p* < 0.001), hypertension (81% vs. 74%, *p* = 0.004), ischaemic cardiomyopathy (82% vs. 70%, *p* < 0.001) and atrial fibrillation (58% vs. 40%, *p* < 0.001), and more frequently reported a hospitalization for heart failure within the prior 12 months before enrolment (40% vs. 29%, *p* < 0.001). Baseline systolic blood pressure, body mass index, and mean haemoglobin levels were similar between groups. While baseline ferritin levels were higher in men (78 ± 59 μg/L in men vs. 63 ± 45 μg/L in women, *p* < 0.001), baseline transferrin saturation was higher in women (18 ± 9 μg/L in men vs. 19 ± 9 μg/L in women, *p* = 0.002). Women assigned to placebo had higher N‐terminal pro‐B‐type natriuretic peptide levels compared to those assigned to ferric carboxymaltose, while the opposite was true for men. Rates of heart failure medication were comparable in women and men, with slightly higher use of sodium–glucose cotransporter 2 (SGLT2) inhibitors and angiotensin receptor–neprilysin inhibitors (ARNI; sacubitril/valsartan) in men.

**Table 1:** Baseline characteristics by sex and treatment group

	Women		Men	
	Placebo (*n* = 169)	FCM (*n* = 199)	Placebo (*n* = 378)	FCM (*n* = 359)
Age (years)	69.1 ± 13.4	68.5 ± 12.7	70.0 ± 11.4	71.0 ± 10.6
Diabetes	68 (40.24)	73 (36.68)	187 (49.47)	175 (48.75)
Hypertension	124 (73.37)	148 (74.37)	302 (79.89)	298 (83.01)
Previous MI	73 (43.20)	71 (35.68)	189 (50.00)	193 (53.76)
Previous PCI	74 (43.79)	83 (41.71)	217 (57.41)	194 (54.04)
Previous CABG	17 (10.06)	22 (11.06)	79 (20.90)	89 (24.79)
Previous stroke or TIA	27 (15.98)	24 (12.06)	68 (17.99)	58 (16.16)
History of atrial fibrillation or flutter	71 (42.01)	78 (39.20)	223 (58.99)	206 (57.38)
Body mass index (kg/m^2^)	28.34 ± 6.53	27.87 ± 6.52	28.07 ± 5.03	28.16 ± 5.26
Ischaemic cause of cardiomyopathy	122 (72.19)	135 (67.84)	308 (81.48)	293 (81.62)
NYHA class				
II	122 (72.19)	147 (73.87)	237 (62.70)	222 (61.84)
III	47 (27.81)	52 (26.13)	137 (36.24)	134 (37.33)
IV	–	–	3 (0.79)	1 (0.28)
Heart failure hospitalization within previous 12 months	49 (28.99)	59 (29.65)	160 (42.33)	134 (37.33)
Systolic BP (mmHg)	119.01 ± 17.97	121.01 ± 18.80	118.66 ± 18.45	120.28 ± 19.00
NT‐proBNP (pg/ml)	4484 ± 7317	3007 ± 5285	3865 ± 5467	5049 ± 7709
6‐min walk test distance (m)	281.7 ± 101.5	278.9 ± 103.0	304.7 ± 96.5	320.7 ± 91.1
eGFR (ml/min/1.73 m^2^)	61.34 ± 23.51	62.71 ± 23.73	59.42 ± 22.82	58.71 ± 23.06
EQ‐5D	0.82 ± 0.18	0.78 ± 0.21	0.82 ± 0.20	0.83 ± 0.21
Presence of pacemaker, implantable defibrillator or resynchronization device	73 (43.20)	81 (40.70)	207 (54.76)	185 (51.53)
Heart failure therapy				
ACEI	78 (46.15)	87 (43.72)	137 (36.24)	153 (42.62)
ARB	27 (15.98)	44 (22.11)	63 (16.67)	56 (15.60)
ARNI (sacubitril/valsartan)	59 (34.91)	63 (31.66)	160 (42.33)	137 (38.16)
Beta‐blocker	158 (93.49)	176 (88.44)	354 (93.65)	328 (91.36)
MRA	125 (73.96)	136 (68.34)	268 (70.90)	250 (69.64)
SGLT2 inhibitor	31 (18.34)	42 (21.11)	100 (26.46)	88 (24.51)
Diuretics	135 (79.88)	160 (80.40)	310 (82.01)	301 (83.84)
Laboratory measurements				
Haemoglobin (g/dl)	12.43 ± 1.15	12.42 ± 1.15	12.44 ± 1.13	12.54 ± 1.14
Ferritin (μg/L)	64.18 ± 46.78	61.96 ± 44.27	78.15 ± 62.32	77.74 ± 54.89
Iron (μg/dl)	69.72 ± 29.06	70.95 ± 33.19	63.83 ± 30.79	65.97 ± 32.36
Transferrin (mg/dl)	269.18 ± 51.45	269.84 ± 57.05	268.35 ± 53.17	265.31 ± 51.14
Transferrin saturation (%)	18.91 ± 8.66	19.50 ± 9.84	17.43 ± 9.04	18.07 ± 8.98

Values are given as mean ± standard deviation, or *n* (%).

ACEI, angiotensin‐converting enzyme inhibitor; ARB, angiotensin receptor blocker; ARNI, angiotensin receptor–neprilysin inhibitor; BP, blood pressure; CABG, coronary artery bypass graft; eGFR, estimated glomerular filtration rate; EQ‐5D, EuroQol‐5 Dimension; FCM, ferric carboxymaltose; MI, myocardial infarction; MRA, mineralocorticoid receptor antagonist; NT‐proBNP, N‐terminal pro‐B‐type natriuretic peptide; NYHA, New York Heart Association; PCI, percutaneous coronary intervention; SGLT2, sodium–glucose cotransporter 2 inhibitor; TIA, transient ischaemic attack.

### Primary outcomes

As shown in *Table* *2*, the absolute rates (per 100 person‐years) of the primary endpoints in ferric carboxymaltose and placebo were lower in women than men, but the relative risk reduction with ferric carboxymaltose was much more unfavourable in women.

**Table 2:** Primary, secondary and safety endpoints

Endpoint	Women				Men				Interaction	
	FCM (*n* = 199)	Placebo (*n* = 169)	Estimate (95% CI)	*p*‐value	FCM (*n* = 359)	Placebo (*n* = 378)	Estimate (95% CI)	*p*‐value	Estimate (95% CI)	*p*‐value
Primary endpoints										
Time‐to‐first event of cardiovascular death or heart failure hospitalization (rate per 100 patient‐years)	33 (10.1)	25 (9.0)	1.07^a^ (0.63–1.82)	0.80	108 (21.0)	141 (29.2)	0.74^a^ (0.57–0.95)	0.016	0.64 (0.36–1.14)	0.13
Total (first and recurrent) heart failure hospitalizations) (rate per 100 patient‐years)	53 (14.3)	39 (12.6)	1.06^b^ (0.55–2.05)	0.86	211 (33.5)	281 (43.3)	0.79^b^ (0.58–1.08)	0.136	0.68 (0.33–1.41)	0.30
Time‐to‐first event of cardiovascular death or heart failure hospitalization in patients with transferrin saturation <20% (rate per 100 patient‐years)	22 (11.1)	15 (8.7)	1.21^a^ (0.62–2.36)	0.58	81 (23.3)	113 (34.5)	0.73^a^ (0.55–0.97)	0.028	0.55 (0.27–1.14)	0.104
Secondary endpoints										
Change in NYHA functional class from baseline to 12 months (points)	–	–	0.522^c^ (0.141–1.929)	0.33	–	–	0.748^c^ (0.353–1.583)	0.45	0.901 (0.238–3.418)	0.88
Change in EQ‐5D score from baseline to 12 months (points)	0.05 ± 0.15	−0.03 ± 0.18	0.055^d^ (0.013–0.098)	0.011	0.01 ± 0.19	−0.01 ± 0.20	0.019^d^ (−0.010–0.049)	0.20	0.036 (−0.015–0.087)	0.16
Change in 6‐min walk distance from baseline to 12 months (m)	33.5 ± 82.1	22.5 ± 83.1	+11.9^d^ (−7.9–31.7)	0.24	23.0 ± 96.8	18.26 ± 85.8	+8.0^d^ (−7.5–23.5)	0.31	+5.7 (−19.6–31.1)	0.66
Patient‐reported global assessment of well‐being score during follow‐up until 12 months (points)	–	–	0.143^c^ (0.072–0.287)	<0.001	–	–	0.341^c^ (0.214–0.543)	<0.001	0.538 (0.329–0.881)	0.014
Safety endpoints										
All‐cause mortality (rate per 100 patient‐years)	25 (6.1)	16 (4.6)	1.46^a^ (0.78–2.76)	0.24	79 (10.6)	95 (12.6)	0.86^a^ (0.64–1.16)	0.33	0.59 (0.29–1.17)	0.13
Cardiovascular mortality (rate per 100 patient‐years)	9 (6.7)	8 (6.2)	0.92^a^ (0.35–2.38)	0.86	45 (7.7)	57 (9.7)	0.80^a^ (0.54–1.18)	0.25	0.80 (0.29–2.25)	0.68

EQ‐5D, EuroQol‐5 Dimension; FCM, ferric carboxymaltose; NYHA, New York Heart Association.

^a^ Hazard ratio.

^b^ Rate ratio.

^c^ Odds ratio.

^d^ Mean difference.

**Figure 2:**
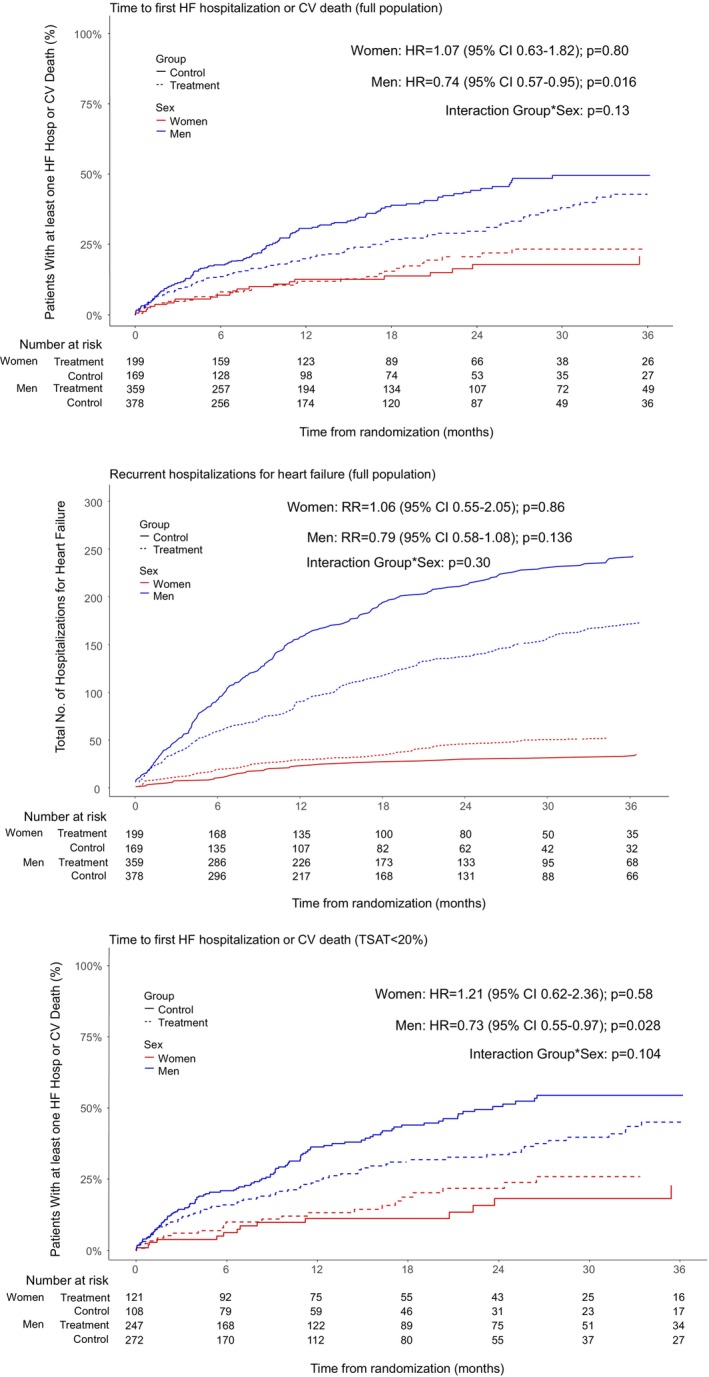
Cumulative incidence of the primary endpoints in women and men. CI, confidence interval; CV, cardiovascular; HF, heart failure; HR, hazard ratio; RR, rate ratio; TSAT, transferrin saturation.

In women, the first primary endpoint of time‐to‐first cardiovascular death or heart failure hospitalization occurred in 33 patients in the treatment group compared with 25 in the placebo group (HR 1.07; 95% CI 0.63–1.82, *p* = 0.80) (*Figure* *2*). The second primary endpoint of total heart failure hospitalizations occurred in 53 women in the treatment group compared with 39 in the placebo group (RR 1.06; 95% CI 0.55–2.05, *p* = 0.86). The third primary endpoint of time to cardiovascular death or first heart failure hospitalization in patients with transferrin saturation <20% occurred in 22 women in the treatment group compared with 15 in the placebo group (HR 1.21; 95% CI 0.62–2.36, *p* = 0.58).

In men, the first primary endpoint of time‐to‐first event of cardiovascular death or heart failure hospitalization occurred in 108 patients in the treatment group compared with 141 in the placebo group (HR 0.74; 95% CI 0.57–0.95, *p* = 0.016) (*Table* *2*; *Figure* *2*). The second primary endpoint of total heart failure hospitalizations occurred in 211 men in the treatment group compared with 281 in the placebo group (RR 0.79; 95% CI 0.58–1.08, *p* = 0.136). The third primary endpoint of time‐to‐first cardiovascular death or heart failure hospitalization in patients with transferrin saturation <20% occurred in 81 men in the treatment group compared with 113 in the placebo group (HR 0.73; 95% CI 0.55–0.97, *p* = 0.028).

The *p*‐value for a treatment‐by‐sex interaction reached borderline significance for two of the three primary endpoints (*p* for interaction 0.13, 0.30, and 0.104, respectively).

### Secondary outcomes

**Figure 3:**
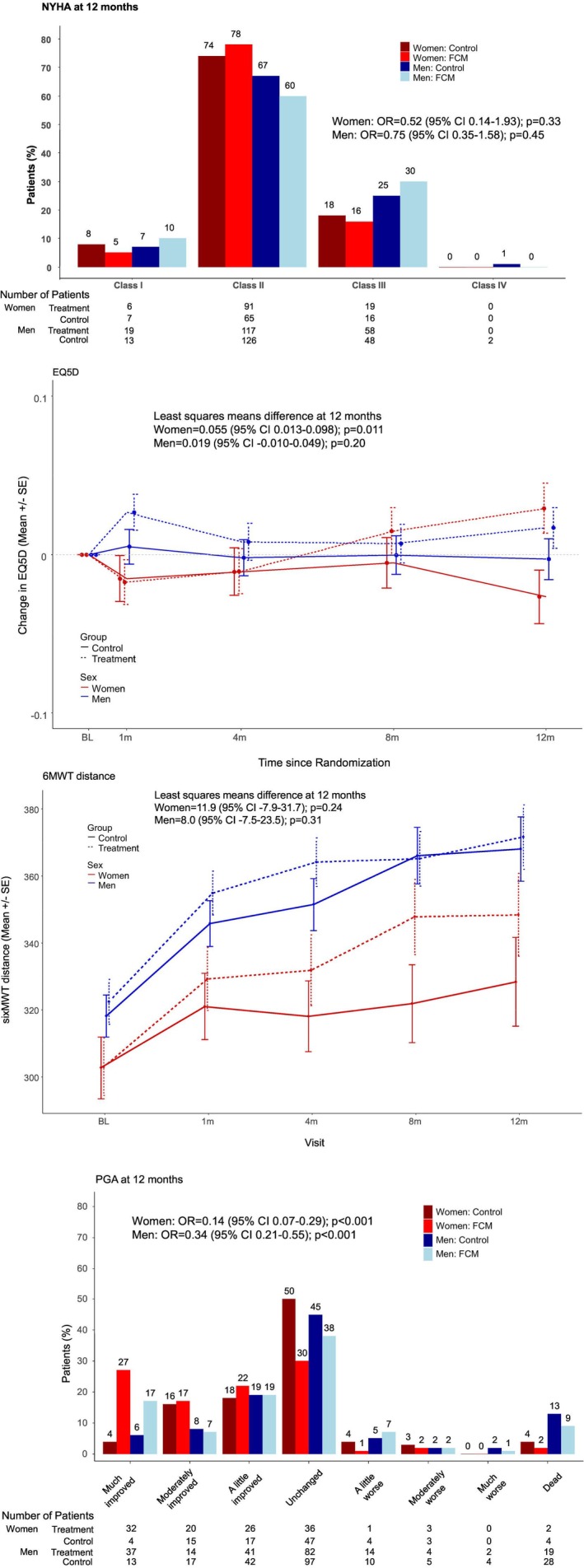
Secondary endpoints in women and men. (*A*) Change in New York Heart Association (NYHA) functional class from baseline to 12 months. (*B*) Change in EuroQol‐5 Dimension (EQ‐5D) score from baseline to 12 months. (*C*) Change in 6‐min walk test (6MWT) distance from baseline to 12 months. (*D*) Change in self‐reported patient global assessment (PGA) score from baseline to 12 months. CI, confidence interval; FCM, ferric carboxymaltose; OR, odds ratio; SE, standard error.

The secondary endpoint analyses were more favourable in women (*Table* *2*; *Figure* *3*). In women, there was an improvement in the EQ‐5D score from baseline to 12 months in the treatment group compared with the placebo group (mean difference: +0.055; 95% CI 0.013–0.098, *p* = 0.011). There was also an improvement in the mean patient‐reported global assessment of well‐being score at 12 months in women in the treatment group versus the placebo group (odds ratio 0.14; 95% CI 0.07–0.29, *p* < 0.001). The mean change from baseline to 12 months in the 6‐min walk test in women was 33.5 ± 82.1 m in the treatment group versus 22.5 ± 83.1 in the placebo group (mean difference: +11.9; 95% CI −7.9–31.7, *p* = 0.24). Finally, the change in NYHA functional class in women was comparable between the treatment and placebo groups (odds ratio 0.52; 95% CI 0.14–1.93, *p* = 0.33).

In men, the improvement in the EQ‐5D score from baseline to 12 months in the treatment group compared with the placebo group did not reach statistical significance (mean difference: +0.019; 95% CI −0.010–0.049, *p* = 0.20). There was a significant improvement in the mean patient‐reported global assessment of well‐being score at 12 months in men in the treatment group versus the placebo group (odds ratio 0.34; 95% CI 0.21–0.54, *p* < 0.001). The mean change from baseline to 12 months in the 6‐min walk test in men was 23.0 ± 96.8 m in the treatment group versus 18.3 ± 85.8 in the placebo group (mean difference: +8.0; 95% CI −7.5–23.5, *p* = 0.31). Finally, in men the change in NYHA functional class was comparable between the treatment and placebo groups (odds ratio 0.75; 95% CI 0.35–1.58, *p* = 0.45). The *p*‐value for a treatment‐by‐sex interaction was 0.16 for the EQ‐5D endpoint, 0.014 for the patient‐reported global assessment of well‐being score endpoint, 0.66 for the 6‐min walk test endpoint, and 0.88 for the change in NYHA functional class endpoint.

### Safety outcomes

The overall incidence of investigator‐reported AEs, serious AEs (SAEs), and AEs leading to study discontinuation or drug withdrawal were similar in both groups in men, but not in women (online supplementary *Table* *S2*). In women, rates of AEs and SAEs coded as ‘cardiac disorders’ and ‘infections and infestations’ were clearly higher in the treatment groups. The frequency of patients with at least one SAE in women was similar in the treatment (*n* = 75, 37.7%) and placebo groups (*n* = 60, 35.5%; Chi‐squared test, *p* = 0.75; data not shown). In men, the frequency of patients with at least one SAE was higher than in women but similar in the treatment (*n* = 194, 54.0%) and placebo groups (*n* = 213, 56.4%; Chi‐squared test, *p* = 0.58; data not shown).

**Figure 4:**
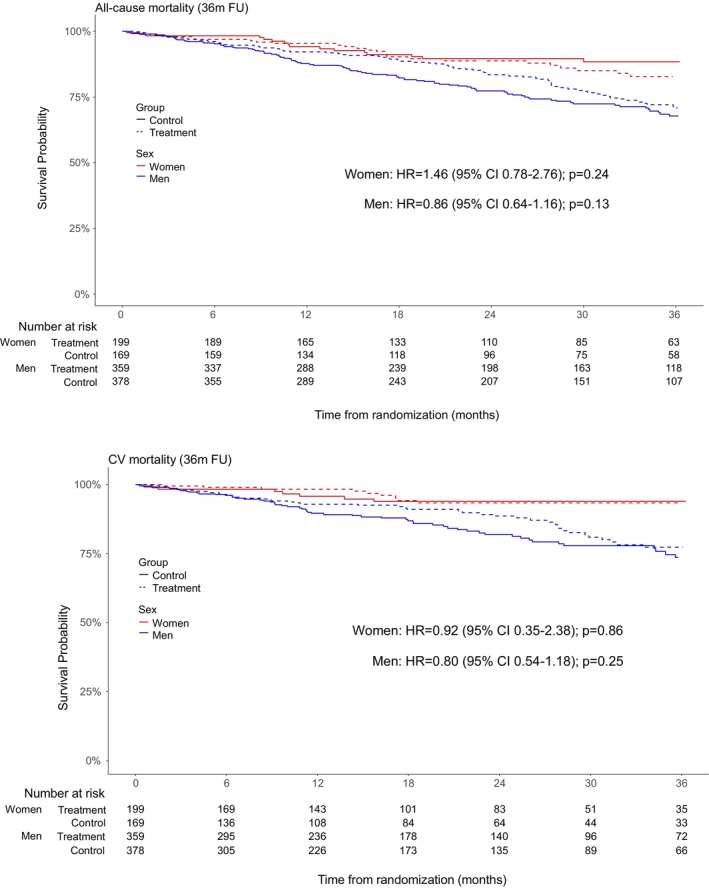
Safety endpoints in women and men. (*A*) All‐cause mortality within 36 months of follow‐up (FU). (*B*) Cardiovascular (CV) mortality within 36 months of FU. CI, confidence interval; HR, hazard ratio.

In women, the total number of deaths due to any cause within 36 months was 25 in the treatment group and 16 in the placebo group (HR 1.46; 95% CI 0.78–2.76, *p* = 0.24), thereby suggesting an increased mortality risk under iron supplementation (*Table* *2*; *Figure* *4*). The total number of deaths due to cardiovascular cause within 36 months in women was 9 in the treatment group and 8 in the placebo group (HR 0.92; 95% CI 0.35–2.38, *p* = 0.86).

In men, the total number of deaths due to any cause within 36 months was 79 in the treatment group and 95 in the placebo group (HR 0.86; 95% CI 0.64–1.16, *p* = 0.33), thereby suggesting a beneficial effect of iron supplementation. The total number of deaths due to cardiovascular cause within 36 months in men was 45 in the treatment group and 57 in the placebo group (HR 0.80; 95% CI 0.54–1.18, *p* = 0.25). The *p*‐value for a treatment‐by‐sex interaction was 0.13 for all‐cause mortality, and thereby borderline not significant, and 0.68 for cardiovascular mortality.

**Figure 5:**
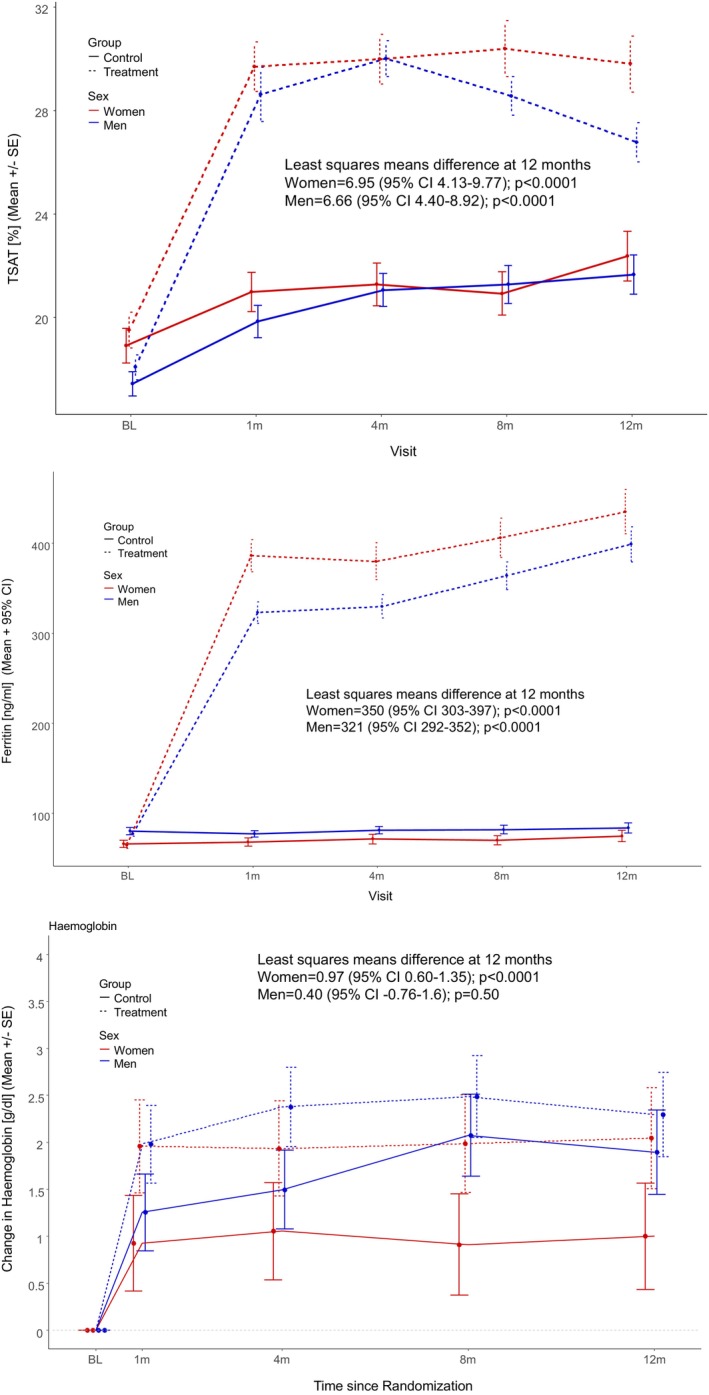
Safety endpoints in women and men. BL, baseline; CI, confidence interval; SE, standard error; TSAT, transferrin saturation.

Changes in transferrin saturation, serum ferritin, and haemoglobin from baseline to month 12 are shown in *Figure* *5*. While increase in transferrin saturation and serum ferritin happened in both, men and women, increase in haemoglobin reached significance in women only (women: least squares mean difference at 12 months between treatment groups 0.97 [95% CI 0.60–1.35]; *p* < 0.0001; men: least squares mean difference at 12 months between treatment groups 0.40 [95% CI 0.76–1.6]; *p* = 0.50).

## Discussion

Despite the well‐established benefits of intravenous iron supplementation in patients with heart failure and iron deficiency, data on sex‐specific outcomes remain rudimentary. Prior studies such as AFFIRM‐AHF and HEART‐FID suggested potential differential effects of iron therapy between women and men, but they lacked comprehensive sex‐stratified analyses. The FAIR‐HF2 trial provides new insights into the sex‐specific efficacy and safety of intravenous ferric carboxymaltose in heart failure with reduced ejection fraction and iron deficiency, highlighting significant differences in outcomes between women and men.

The primary findings of this study suggest a stark contrast in the response to intravenous ferric carboxymaltose between women and men. While men receiving ferric carboxymaltose experienced a clinically relevant reduction in cardiovascular death and heart failure hospitalizations (HR 0.74, 95% CI 0.57–0.95, *p* = 0.016), women did not derive similar benefits (HR 1.07, 95% CI 0.63–1.82, *p* = 0.80). Furthermore, the observed interaction *p*‐values indicate a potential sex–treatment interaction trend, warranting further exploration. This finding is somehow surprising: since women were significantly healthier and also had much less hospitalizations within the previous 12 months before randomization, one would intuitively expect rather better outcomes than in men.

Conversely, secondary endpoints assessing symptomatic and functional improvement favoured women. Women receiving ferric carboxymaltose demonstrated greater improvements in EQ‐5D scores and self‐reported global assessment of well‐being than men. This finding suggests that despite the lack of prognostic benefit, women may still experience meaningful symptomatic relief and improved quality of life. This stronger efficacy in symptomatic endpoints in women is probably due to a differential effect on haemoglobin: as shown in *Figure* *5*, the difference between treatment groups in women from baseline to month 12 is 1 g/dl (*p* < 0.001), while there is no significant difference between the treatment groups in men (*p* = 0.50). The disparity between primary and secondary outcomes is further complicated by the safety findings, where a trend towards increased all‐cause mortality was observed in women (HR 1.46; 95% CI 0.78–2.76, *p* = 0.24), compared to a neutral or even beneficial trend in men (HR 0.86; 95% CI 0.64–1.16, *p* = 0.33).

Our findings are in line with a recent meta‐analysis, which summarizes the efficacy and safety of intravenous iron from six trials (FAIR‐HF, CONFIRM‐HF, AFFIRM‐AHF, IRONMAN, HEART‐FID, and FAIR‐HF2) including 7175 patients: patients assigned to intravenous iron, as compared to those assigned to placebo, had lower rates for the composite endpoint of recurrent heart failure hospitalizations and cardiovascular mortality (RR 0.81; 95% CI 0.63–0.97). The subgroup analyses revealed no significant differences in the effect of intravenous iron on the primary endpoint, with the exception of sex.^12^ There was a significant interaction for sex (ratio of RR 1.40; 95% CI 1.05–1.86), with women on average showing no benefit (RR 0.98; 95% CI 0.75–1.26).^12^

This new finding was not indicated in previous large‐scale prospective biomarker studies in secondary prevention cohorts.^13^, ^14^, ^15^, ^16^ While these studies adjusted for sex in their prediction models, they do not provide sex‐stratified analyses.^13^, ^14^, ^15^, ^16^ Prospective sex‐specific data in biomarker studies from large‐scale cohorts are only available in the primary prevention setting: the Gutenberg Health Study reports similar predictive value of iron deficiency in 5000 men and women.^17^, ^18^ The HR for the association of absolute iron deficiency with all‐cause mortality during 5‐year follow‐up was 1.97 (95% CI 1.10–3.53; *p* = 0.02) in men and 1.77 (95% CI 1.00–3.14; *p* < 0.01) in women.^17^

Several potential biological mechanisms, like sex‐specific differences in iron metabolism, erythropoiesis, inflammation, and hormonal regulation, may explain the differential effects of intravenous ferric carboxymaltose in this population of overwhelmingly post‐menopausal women and men. It is well known that oestrogen manipulates intracellular iron metabolism.^4^ Moreover, elevated levels of oestrogen are associated with increased systemic iron availability, which potentially might alter the therapeutic response to intravenous iron.^4^ This has been attributed to the ability of oestrogen to suppress hepcidin synthesis, maintain ferroportin integrity, and enhance iron release from iron‐absorbing duodenal enterocytes and iron‐storing macrophages and hepatocytes.^4^, ^19^ Yang and colleagues examined the effects of 17β‐estradiol (E2) on hepcidin, the key negative regulator of iron absorption from the liver.^20^ They found that transcription of hepcidin was suppressed by E2 treatment in human liver HuH7 and HepG2 cells, and this down‐regulation was blocked by E2 antagonist ICI 182780.^20^ Their data suggest that hepcidin inhibition by E2 is to increase iron uptake, a mechanism to compensate iron loss during menstruation that might, in part, explain why women develop heart diseases later than men during the reproductive phase of their life.^20^ The mean age among women was 69 years at time of randomization, and thereby much higher than the median age at natural menopause of 51.4 years in high‐income countries.^21^ One may hypothesize that severely reduced oestrogen levels in our overwhelmingly post‐menopausal population, which leads to increased hepcidin levels, may alleviate the utilization of the supplemental intravenous iron and thereby explain the differential efficacy in women.^22^ This hypothesis is supported by a study of Hou and coworkers: they showed that oestrogen deficiency induced by ovariectomy in mice resulted in a great increase in hepatic hepcidin levels that led to decreased serum iron levels.^22^

The adverse effect profile of ferric carboxymaltose in FAIR‐HF2 implies some concerns about its optimal treatment regimen in women. The numerically increased mortality in this population of overwhelmingly post‐menopausal women together with the lack of beneficial effect on prognostic endpoints, despite symptomatic and functional improvements, suggests that iron therapy might influence underlying pathophysiological pathways differently in postmenopausal women compared to men. It remains unclear whether these observations are the result of an unfavourable side effect profile in women. In women, the rates of AEs and SAEs coded as ‘cardiac disorders’ and ‘infections and infestations’ were clearly higher in the treatment group. Furthermore, the consequences of the significant increase in haemoglobin values by 1 g/dl in women remains uncertain. In this context, another study has recently given rise to discussion: the study in 11 women and one man showed that myocardial iron uptake from standard doses of ferric carboxymaltose is independent of the canonical pathway of the reticuloendothelial system, and that intravenous iron is delivered into myocardium rapidly via non‐transferrin‐bound iron transporters.^23^ This iron remained in the myocardium in labile form until the follow‐up 6 weeks later. The authors concluded that the accumulation and persistence of myocardial iron in labile form raises the potential for repeated doses to cause toxicity and thereby lead to unfavourable prognostic outcomes. A subsequent study in these patients just recently suggested that myocardial iron intake following intravenous iron therapy with ferric carboxymaltose was sustained at 1 year despite recurrence of iron deficiency.^24^ Due to its specific patient characteristics and multiple methodological shortcomings, the burden of proof of this study is limited, and forthcoming studies need to validate its potential clinical relevance.

The differential effects of ferric carboxymaltose observed in FAIR‐HF2 do not align with findings from other modern heart failure therapies. Sex‐based analyses of SGLT2 inhibitors dapagliflozin and empagliflozin, vericiguat, and ARNI (sacubitril/valsartan) have demonstrated a very homogeneous and consistent efficacy between men and women.^25^, ^26^, ^27^, ^28^ The contrasting findings from FAIR‐HF2 emphasize the need for dedicated follow‐up studies to evaluate the sex‐specific treatment effects of intravenous iron supplementation in heart failure.

### Limitations

Several limitations of the FAIR‐HF2 trial should be acknowledged. First, the median duration of follow‐up was 16.6 months, therefore long‐term safety and efficacy analysis is limited. Second, potential confounders such as differences in baseline comorbidities, medication adherence, and healthcare access may have influenced the results. Nevertheless, since women were significantly healthier and also had much less hospitalizations within the previous 12 months before randomization, one would intuitively have expected better outcomes than in men. Finally, longer follow‐up and mechanistic studies are needed to further elucidate the biological underpinnings of sex differences in response to intravenous iron therapy.

## Conclusions

This pre‐specified analysis in FAIR‐HF2 represents by far the most comprehensive sex‐specific analysis of intravenous iron to date. The findings are unusual in modern heart failure trials with in general similar benefits in men and women, and may indicate a lack of prognostic benefit in women, despite symptomatic and functional improvements, and could therefore implies some concerns regarding the potential for increased mortality in women receiving intravenous iron replenishment. The results are clinically relevant and prompt validation via detailed sex‐specific analyses in the other large outcome trials of intravenous iron supplementation in heart failure.

Our study suggests that the pathophysiology of iron deficiency and heart failure may differ between sexes, and highlights the need for sex‐specific considerations in heart failure management and underscore the importance of further research to refine treatment strategies for iron deficiency in heart failure patients.

## Supporting information


**Appendix S1.** Supporting Information.

## Data Availability

The FAIR‐HF2‐DZHK05 investigators will review proposals for data sharing after the publication of the primary study and key secondary studies. Data underlying the findings of this manuscript may be obtained in accordance with German Centre for Cardiovascular Research and Vifor Pharma's data sharing policy. Enquiries can be made to the corresponding authors.
