# Supplementary material for: Intravenous ferric carboxymaltose in heart failure with iron deficiency (FAIR‐HF2 DZHK05 trial): Sex‐specific outcomes
Source: Eur J Heart Fail. 2025 Jul 31;27(11):2328–42. doi: 10.1002/ejhf.3742 (PMC12765045; doi:10.1002/ejhf.3742)
Supplement: Supplementary file 1 — Appendix S1. Supporting Information. [file EJHF-27-2328-s001.docx]

**Table S1: Baseline Characteristics by Sex**

|  | **Overall**  N=1105 | **Women**  N=368 | **Men**  N=737 | **p-value** |
| --- | --- | --- | --- | --- |
| Age (years) | 69.9 ± 11.7 | 68.7 ± 13.0 | 70.5 ± 11.0 | 0.10 |
| Diabetes (N, %) | 503 (45.52%) | 141 (38.32%) | 362 (49.12%) | <0.001 |
| Hypertension (N, %) | 872 (78.91%) | 272 (73.91%) | 600 (81.41%) | 0.004 |
| Previous MI (N, %) | 526 (47.60%) | 144 (39.13%) | 382 (51.83%) | <0.001 |
| Previous PCI (N, %) | 568 (51.40%) | 157 (42.66%) | 411 (55.77%) | <0.001 |
| Previous CABG (N, %) | 207 (18.73%) | 39 (10.60%) | 168 (22.80%) | <0.001 |
| Previous Stroke or TIA (N, %) | 177 (16.02%) | 51 (13.86%) | 126 (17.10%) | 0.2 |
| History of atrial fibrillation or flutter (N, %) | 578 (52.31%) | 149 (40.49%) | 429 (58.21%) | <0.001 |
| Body Mass Index (kg/m^2^) | 28.1 ± 5.6 | 28.1 ± 6.5 | 28.1 ± 5.1 | 0.4 |
| Ischaemic cause of cardiomyopathy (N, %) | 858 (77.65%) | 257 (69.84%) | 601 (81.55%) | <0.001 |
| NYHA Class II (N, %) | 728 (66.00%) | 269 (73.10%) | 459 (62.45%) | <0.001 |
| NYHA Class III (N, %) | 370 (33.54%) | 99 (26.90%) | 271 (36.87%) |  |
| NYHA Class IV (N, %) | 4 (0.36%) | 0 (0.00%) | 4 (0.54%) |  |
| Heart failure hospitalisation within previous 12 months (N, %) | 402 (36.38%) | 108 (29.35%) | 294 (39.89%) | <0.001 |
| Systolic BP (mmHg) | 119.7 ± 18.6 | 120.1 ± 18.4 | 119.5 ± 18.7 | 0.5 |
| NT-proBNP (pg/mL*) ** | 4,205.87 ± 6,557.2 | 3,711.72 ± 6,363.7 | 4,421.06 ± 6,634.5 | 0.002 |
| Six Minute Walk Test Distance (m) | 298.4 ± 98.8 | 280.2 ± 102.1 | 312.0 ± 94.2 | <0.001 |
| Estimated Glomerular Filtration Rate (mL/min/1.72cm^2^) | 60.1 ± 23.2 | 62.1 ± 23.6 | 59.1 ± 22.9 | 0.063 |
| EQ-5D, mean | 0.82 ± 0.20 | 0.80 ± 0.20 | 0.83 ± 0.21 | 0.001 |
| Presence of pacemaker, implantable defibrillator or resynchronisation device (N, %) | 546 (49.41%) | 154 (41.85%) | 392 (53.19%) | <0.001 |
| **Heart failure therapy** |  |  |  |  |
| ACEI (N, %) | 455 (41.18%) | 165 (44.84%) | 290 (39.35%) | 0.081 |
| ARB (N, %) | 190 (17.19%) | 71 (19.29%) | 119 (16.15%) | 0.2 |
| ARNI (Sacubitril/Valsartan) (N, %) | 419 (37.92%) | 122 (33.15%) | 297 (40.30%) | 0.021 |
| Beta blocker (N, %) | 1,016 (91.95%) | 334 (90.76%) | 682 (92.54%) | 0.3 |
| MRA (N, %) | 779 (70.50%) | 261 (70.92%) | 518 (70.28%) | 0.8 |
| SGLT2 inhibitor (N, %) | 261 (23.62%) | 73 (19.84%) | 188 (25.51%) | 0.036 |
| Diuretics (N, %) | 906 (81.99%) | 295 (80.16%) | 611 (82.90%) | 0.3 |
| **Laboratory measurements, mean (SD)** |  |  |  |  |
| Haemoglobin [g/dL] | 12.47 ± 1.14 | 12.42 ± 1.15 | 12.49 ± 1.13 | 0.4 |
| Ferritin [µg/L] | 72.97 ± 55.12 | 62.98 ± 45.39 | 77.95 ± 58.78 | <0.001 |
| Iron [µg/dL] | 66.71 ± 31.58 | 70.39 ± 31.34 | 64.87 ± 31.56 | <0.001 |
| Transferrin [mg/dL] | 267.77 ± 52.95 | 269.54 ± 54.49 | 266.87 ± 52.18 | 0.6 |
| Transferrin saturation [%] | 18.23 ± 9.13 | 19.23 ± 9.31 | 17.74 ± 9.01 | 0.002 |

Abbreviations:

MI: myocardial infarction; PCI: percutaneous coronary intervention; CABG: coronary artery bypass graft; TIA: transient ischemic attack; NYHA: New York Heart Association; BP: blood pressure; NT-proBNP: N-terminal pro-brain natriuretic peptide; ACEI: angiotensin converting enzyme inhibitor; ARB: angiotensin receptor blocker; ARNI: angiotensin receptor neprilysin inhibitor; MRA: mineralocorticoid receptor antagonist; SGLT-2: sodium-glucose contransporter-2 inhibitor.

**Table S2: Investigator-reported adverse events, serious adverse events, and adverse events leading to study discontinuation or drug withdrawal**

| **Adverse Events** | **female** | | **male** | |
| --- | --- | --- | --- | --- |
| **Characteristic** | **Control**, N = 321 | **Treatment**, N = 504 | **Control**, N = 1,449 | **Treatment**, N = 1,302 |
| Blood and lymphatic system disorders | 14 (4.36%) | 8 (1.59%) | 26 (1.79%) | 20 (1.54%) |
| Cardiac disorders | 61 (19.00%) | 103 (20.44%) | 400 (27.61%) | 321 (24.65%) |
| Congenital, familial and genetic disorders | 1 (0.31%) | 2 (0.40%) | 3 (0.21%) | 2 (0.15%) |
| Ear and labyrinth disorders | 0 (0.00%) | 2 (0.40%) | 10 (0.69%) | 4 (0.31%) |
| Endocrine disorders | 1 (0.31%) | 3 (0.60%) | 11 (0.76%) | 10 (0.77%) |
| Eye disorders | 3 (0.93%) | 2 (0.40%) | 12 (0.83%) | 10 (0.77%) |
| Gastrointestinal disorders | 18 (5.61%) | 37 (7.34%) | 66 (4.55%) | 87 (6.68%) |
| General disorders and administration site conditions | 15 (4.67%) | 28 (5.56%) | 67 (4.62%) | 72 (5.53%) |
| Hepatobiliary disorders | 1 (0.31%) | 3 (0.60%) | 9 (0.62%) | 12 (0.92%) |
| Immune system disorders | 0 (0.00%) | 5 (0.99%) | 2 (0.14%) | 4 (0.31%) |
| Infections and infestations | 39 (12.15%) | 105 (20.83%) | 170 (11.73%) | 184 (14.13%) |
| Injury, poisoning and procedural complications | 23 (7.17%) | 29 (5.75%) | 46 (3.17%) | 49 (3.76%) |
| Investigations | 9 (2.80%) | 19 (3.77%) | 51 (3.52%) | 58 (4.45%) |
| Metabolism and nutrition disorders | 14 (4.36%) | 15 (2.98%) | 82 (5.66%) | 48 (3.69%) |
| Musculoskeletal and connective tissue disorders | 29 (9.03%) | 18 (3.57%) | 43 (2.97%) | 49 (3.76%) |
| Neoplasms benign, malignant and unspecified (incl cysts and polyps) | 8 (2.49%) | 5 (0.99%) | 28 (1.93%) | 28 (2.15%) |
| Nervous system disorders | 22 (6.85%) | 20 (3.97%) | 83 (5.73%) | 55 (4.22%) |
| Product issues | 3 (0.93%) | 4 (0.79%) | 6 (0.41%) | 7 (0.54%) |
| Psychiatric disorders | 3 (0.93%) | 2 (0.40%) | 7 (0.48%) | 8 (0.61%) |
| Renal and urinary disorders | 11 (3.43%) | 22 (4.37%) | 92 (6.35%) | 80 (6.14%) |
| Reproductive system and breast disorders | 0 (0.00%) | 1 (0.20%) | 1 (0.07%) | 8 (0.61%) |
| Respiratory, thoracic and mediastinal disorders | 4 (1.25%) | 25 (4.96%) | 82 (5.66%) | 53 (4.07%) |
| Skin and subcutaneous tissue disorders | 6 (1.87%) | 10 (1.98%) | 36 (2.48%) | 22 (1.69%) |
| Surgical and medical procedures | 19 (5.92%) | 24 (4.76%) | 69 (4.76%) | 64 (4.92%) |
| Vascular disorders | 17 (5.30%) | 12 (2.38%) | 47 (3.24%) | 47 (3.61%) |
|  |  |  |  |  |
| **Serious Adverse Events** | **female** | | **male** | |
| **Characteristic** | **Control**, N = 151 | **Treatment**, N = 189 | **Control**, N = 749 | **Treatment**, N = 661 |
| Blood and lymphatic system disorders | 8 (5.30%) | 2 (1.06%) | 11 (1.47%) | 12 (1.82%) |
| Cardiac disorders | 49 (32.45%) | 71 (37.57%) | 335 (44.73%) | 261 (39.49%) |
| Congenital, familial and genetic disorders | 0 (0.00%) | 1 (0.53%) | 1 (0.13%) | 1 (0.15%) |
| Endocrine disorders | 0 (0.00%) | 1 (0.53%) | 3 (0.40%) | 1 (0.15%) |
| Gastrointestinal disorders | 5 (3.31%) | 11 (5.82%) | 25 (3.34%) | 38 (5.75%) |
| General disorders and administration site conditions | 7 (4.64%) | 14 (7.41%) | 26 (3.47%) | 30 (4.54%) |
| Hepatobiliary disorders | 1 (0.66%) | 1 (0.53%) | 4 (0.53%) | 8 (1.21%) |
| Immune system disorders | 0 (0.00%) | 4 (2.12%) | 1 (0.13%) | 0 (0.00%) |
| Infections and infestations | 11 (7.28%) | 19 (10.05%) | 70 (9.35%) | 84 (12.71%) |
| Injury, poisoning and procedural complications | 10 (6.62%) | 9 (4.76%) | 14 (1.87%) | 17 (2.57%) |
| Investigations | 5 (3.31%) | 6 (3.17%) | 12 (1.60%) | 10 (1.51%) |
| Metabolism and nutrition disorders | 0 (0.00%) | 1 (0.53%) | 11 (1.47%) | 11 (1.66%) |
| Musculoskeletal and connective tissue disorders | 9 (5.96%) | 1 (0.53%) | 6 (0.80%) | 9 (1.36%) |
| Neoplasms benign, malignant and unspecified (incl cysts and polyps) | 6 (3.97%) | 5 (2.65%) | 25 (3.34%) | 19 (2.87%) |
| Nervous system disorders | 6 (3.97%) | 7 (3.70%) | 39 (5.21%) | 22 (3.33%) |
| Product issues | 3 (1.99%) | 4 (2.12%) | 6 (0.80%) | 4 (0.61%) |
| Renal and urinary disorders | 6 (3.97%) | 8 (4.23%) | 47 (6.28%) | 46 (6.96%) |
| Respiratory, thoracic and mediastinal disorders | 2 (1.32%) | 7 (3.70%) | 31 (4.14%) | 18 (2.72%) |
| Skin and subcutaneous tissue disorders | 1 (0.66%) | 0 (0.00%) | 8 (1.07%) | 3 (0.45%) |
| Surgical and medical procedures | 12 (7.95%) | 13 (6.88%) | 44 (5.87%) | 38 (5.75%) |
| Vascular disorders | 10 (6.62%) | 4 (2.12%) | 23 (3.07%) | 21 (3.18%) |
| Ear and labyrinth disorders | 0 (0.00%) | 0 (0.00%) | 1 (0.13%) | 0 (0.00%) |
| Eye disorders | 0 (0.00%) | 0 (0.00%) | 4 (0.53%) | 3 (0.45%) |
| Psychiatric disorders | 0 (0.00%) | 0 (0.00%) | 2 (0.27%) | 3 (0.45%) |
| Reproductive system and breast disorders | 0 (0.00%) | 0 (0.00%) | 0 (0.00%) | 2 (0.30%) |

**Table S3: Primary endpoints (FU cut at 12 months)**

|  | **Women** | | | | **Men** | | | | **Interaction** | |
| --- | --- | --- | --- | --- | --- | --- | --- | --- | --- | --- |
| **Endpoint** | FCM (N=199) | Placebo (N=169) | Estimate  (95% CI) | P-Value | FCM (N=359) | Placebo (N=378) | Estimate  (95% CI) | P-Value | Estimate (95% CI) | P-value |
| **Primary Endpoints (FU cut at 12 months)** | | | | | | | | |  |  |
| Time to first event of cardiovascular death or heart failure hospitalisation (rate per 100 patient years) | 21 (6.2) | 18 (6.3) | 0.98*  (0.52 – 1.84) | 0.94 | 63 (11.4) | 98 (18.0) | 0.67*  (0.48 – 0.91) | 0.011 | 0.67  (0.33-1.35) | 0.257 |
| Total (first and recurrent) heart failure hospitalisations (rate per 100 patient years) | 29 (17.4) | 23 (16.9) | 1.01^**^  (0.43 – 2.36) | 0.98 | 90 (31.2) | 158 (53.4) | 0.60^**^  (0.42 – 0.86) | 0.005 | 0.57  (0.23-1.41) | 0.228 |
| Time to first event of cardiovascular death or heart failure hospitalisation in patients with transferrin saturation less than 20% (rate per 100 patient years) | 14 (6.7) | 10 (5.5) | 1.22*  (0.54 – 2.78) | 0.627 | 52 (14.0) | 82 (21.8) | 0.69*  (0.49 – 0.98) | 0.038 | 0.56  (0.23-1.35) | 0.19 |

Abbreviations

FCM: Ferric carboxymaltose; FU: Follow-Up.

**Table S4: Total amount of ferric carboxymaltose received and number of discontinuations**

|  | **Women** | | | | **Men** | | | |
| --- | --- | --- | --- | --- | --- | --- | --- | --- |
|  | FCM (N=199) | | | Placebo (N=169) | FCM (N=359) | | | Placebo (N=378) |
|  | Year 1 | Year 2 | Year 3 |  | Year 1 | Year 2 | Year 3 |  |
| **Total amount of FCM received [mg], mean (SD)** | 1921 (470) | 895 (422) | 745 (435) | n/a | 2117 (426) | 942 (423) | 752 (445) | n/a |
| **Number of discontinuations** | 60 | | | 54 | 130 | | | 152 |

Abbreviations:

FCM: Ferric carboxymaltose; N: Number; SD: Standard deviation.

**Table S5: Results for primary endpoint adjusted for conventional cardiovascular risk factors**

|  | **Women** | | | | **Men** | | | | **Interaction** | |
| --- | --- | --- | --- | --- | --- | --- | --- | --- | --- | --- |
| **Endpoint** | FCM (N=199) | Placebo (N=169) | Estimate  (95% CI) | P-Value | FCM (N=359) | Placebo (N=378) | Estimate  (95% CI) | P-Value | Estimate (95% CI) | P-value |
| **Primary Endpoints (further adjusted for differences in patient characteristics, i. e. diabetes, etiology, previous HF hospitalization within 12 month prior to baseline)** | | | | | | | | |  |  |
| Time to first event of cardiovascular death or heart failure hospitalisation (rate per 100 patient years) | 33 (10.1) | 25 (9.0) | 1.01*  (0.59 – 1.73) | 0.97 | 108 (21.0) | 141 (29.2) | 0.76*  (0.59 – 0.97) | 0.029 | 0.70  (0.39-1.25) | 0.224 |
| Total (first and recurrent) heart failure hospitalisations (rate per 100 patient years) | 53 (14.3) | 39 (12.6) | 1.01^**^  (0.52 – 1.94) | 0.98 | 211 (33.5) | 281 (43.3) | 0.81^**^  (0.59 – 1.11) | 0.184 | 0.77  (0.37-1.59) | 0.474 |
| Time to first event of cardiovascular death or heart failure hospitalisation in patients with transferrin saturation less than 20% (rate per 100 patient years) | 22 (11.1) | 15 (8.7) | 1.23*  (0.62 – 2.42) | 0.56 | 81 (23.3) | 113 (34.5) | 0.74*  (0.56 – 0.99) | 0.041 | 0.59  (0.28-1.21) | 0.15 |

Abbreviations:

FCM: Ferric carboxymaltose; N: Number; SD: Standard deviation.
